# BSSRO Improves Mandibular Morphology Mainly through Correction of Body Length and Volume in Patients with Asymmetric Mandibular Prognathism

**DOI:** 10.3390/jcm11237131

**Published:** 2022-11-30

**Authors:** Yanfei Liu, Yunfeng Li

**Affiliations:** State Key Laboratory of Oral Diseases, National Clinical Research Center for Oral Diseases, Orthognathic & TMJ Surgery Center, West China Hospital of Stomatology, Sichuan University, Chengdu 610041, China

**Keywords:** orthognathic surgery, mandibular deviation, BSSRO, condyle, recurrence

## Abstract

Purpose: The purpose of this study is to analyze the change and stability of mandibular morphology in patients with asymmetric mandibular prognathism after bilateral sagittal split ramus osteotomy (BSSRO). Methods: We retrospectively analyzed fifty patients with asymmetric mandibular prognathism from the West China Hospital of Stomatology, Sichuan University, between January 2018 to March 2021. The spiral CT data before surgery, within two weeks after surgery, and at more than six months after surgery of each patient were collected. According to the deflection direction of the chin, the bilateral mandibles were defined as the long side and the short side. The morphological data of the bilateral condyle, the mandibular ramus, and the mandibular body were analyzed to determine the effect and stability of BSSRO on asymmetric mandibular prognathism. Results: It was found that the long-side mandible had greater condylar volume and diameter, mandibular ramus height and volume, and mandibular body length and volume (*p* < 0.05) before surgery. After surgery, the volume of the mandibular ramus increased, while the length and volume of the mandibular body decreased (*p* < 0.05) at the long side of the mandible; the morphological changes of the mandibular ramus and body at the short side of the mandible were not statistically significant (*p* > 0.05). When comparing the long and short sides of the mandible, the long side still had greater height and volume of the mandibular ramus (*p* < 0.01). The volume difference of the mandibular body from the two sides was corrected (*p* > 0.05), and the length difference of the mandibular body from the two sides was overcorrected (*p* < 0.05). At more than six months after surgery, the volume of the mandibular ramus and body increased, while their height decreased at the long side of the mandible (*p* < 0.05). For the other side, or the short side, the volume of the ramus and body increased, too. However, their height decreased (*p* < 0.01). Conclusion: The results of this study suggested good effect and stability of BSSRO on asymmetric mandibular prognathism, except for the correction of ramus height and volume.

## 1. Introduction

One etiological factor of dentofacial deformities is abnormal development of the underlining jaws, which manifests as occlusal dysfunction and abnormal maxillofacial appearance, also known as skeletal malocclusion [1,2]. According to existing statistics, 40% of the population suffer from malocclusion (faulty contact between the upper and lower teeth when the jaw is closed), of which about 5% are skeletal malocclusion. A retrospective study conducted by Chew et al showed that 48% of skeletal class III patients were accompanied by facial asymmetry, usually diagnosed as asymmetric mandibular prognathism [3]. The most commonly used orthognathic procedure for asymmetric mandibular prognathism is bilateral sagittal split ramus osteotomy (BSSRO) [4], which corrects the mandibular protrusion and deviation through the retraction and rotation of the distal bone segment with dentition.

In view of the strong demand for postoperative symmetry in such patients, it is of great clinical significance to study the change of the mandibular symmetry after BSSRO. In addition, due to the effect of soft tissues, such as muscles, patients with asymmetric mandibular prognathism show a certain tendency to recur after surgery, which may be manifested as changes in the position of the condyle, or changes in the morphology of the mandibular ramus and body. The changing pattern of the position of the condyle has been extensively studied. Thus, this study mainly investigates the changeable rules of the mandibular morphology after BSSRO in patients with asymmetric mandibular prognathism [5,6].

Traditional 2D X-ray has image magnification, inter-overlapping, fixed-point error, and other distortions. Compared to 2D radiographs, 3D cephalometric measurement can more accurately locate anatomies and evaluate complex skull structures. Thus, CT scans have been advised to provide more accurate and detailed information for the diagnosis and treatment plan of asymmetric mandibular prognathism [7,8].

In this study, we aim to find the changeable rules and stability of mandibular morphology in patients with asymmetric mandibular prognathism after BSSRO.

## 2. Patients and Methods

### 2.1. Patients

Data from patients who were diagnosed with asymmetric mandibular prognathism at the department of Orthognathic and Temporomandibular joint (TMJ) Surgery, West China Hospital Stomatology, Sichuan University, during January 2018 to March 2021 were collected. All patients received combined orthodontic and orthognathic treatment. All patients received BSSRO under general anesthesia, and some underwent genioplasty at the same time. The doctors used the internal approach, and titanium plates and screws were used for solid internal fixation. Intermaxillary elastic traction and fixation were maintained for 1–2 weeks from the third day after operation. The study protocol was approved by the West China Hospital of Stomatology Institutional Review Board (WCSHIRB). 

The inclusion criteria were as follows:

(1) Adult patients diagnosed with asymmetric mandibular prognathism;
(2) ANB angle was less than 0 degree;
(3) The distance between the submental point and the midsagittal plane on the 3D reconstruction model was ≥4 mm;
(4) Patients were treated with orthodontic appliance and orthognathic surgery;
(5) Patients who accepted BSSRO with or without genioplasty.

The exclusion criteria were as follows:

(1) Patients with maxillofacial trauma or jaw deformity secondary to cleft lip and palate;
(2) Patients with condylar tumor;
(3) Patients with systemic diseases;
(4) Patients with a history of camouflaged orthognathic surgery;
(5) Patients who underwent maxillary orthognathic surgery at the same time (some studies have shown that the change of the condyle and the ramus angle after single mandibular surgery is different from that of bimaxillary surgery [9]. In order to minimize this system error, we chose patients who had mandibular surgery only).

### 2.2. Data Collection and Processing

During the treatment, the patients with asymmetric mandibular prognathism received at least 3 CT scans. The first checkup (T0) occurred after presurgical orthodontic treatment and before orthognathic surgery, the purpose of which was for virtual surgical planning. The second checkup (T1) occurred within 2 weeks after surgery to confirm postoperative mandible position and occlusal relationship. The third checkup (T2) occurred more than half a year after surgery, and its purpose was to evaluate the healing of bone and whether the asymmetry recurred or not. Thus, maxillofacial spiral CT data (Philips MX16 EVO, Aurora, IL, USA, kV:120, mAs: 230, inversion time: 19,663 ms) at T0, T1, and T2 of each patient were collected. The Digital Imaging and Communications in Medicine (DICOM) data were imported into mimics 21.0 software (Materialises Interactive Medical Image Control System, Leuven, Belgium), and the bone tissue was three-dimensionally reconstructed. The mandible was completely separated using the Split Mask function of the software.

### 2.3. Selection of Marker Points and Reference Planes

According to the deflection direction of the chin, the bilateral mandibles were defined as the long side and the short side. For instance, when the chin was inclined to the left, the left mandible was the short side, and the right mandible was the long side. Eleven jawbone landmarks and three reference planes were selected according to previous studies (Table 1) [10,11,12,13].

### 2.4. Measurement Items (Figure 1 and Figure 2)

(1) Condyle volume: the volume of the part above the plane which is parallel to the HF and passes through the lowest point of the sigmoid notch.
(2) Medial–lateral diameter of the condyle: the distance from the innermost point to the outermost point of the condyle.
(3) Anterior–posterior diameter of the condyle: the distance from the most anterior point to the last point of the condyle.
(4) Ramus height: the distance from Co to Go-inf.
(5) Ramus volume: the volume of the part of the mandible above the plane established by J-lat, J-med, and Go-inf.
(6) Body volume: after making a plane parallel to the MSP through the MF, the body volume is the volume of the distal part of this plane, excluding the ramus volume.
(7) Body length: the distance from Go-post to MF.
(8) Body height: the vertical distance between MP and the margin of the distal alveolar bone of the first molar.

**Figure 1:**
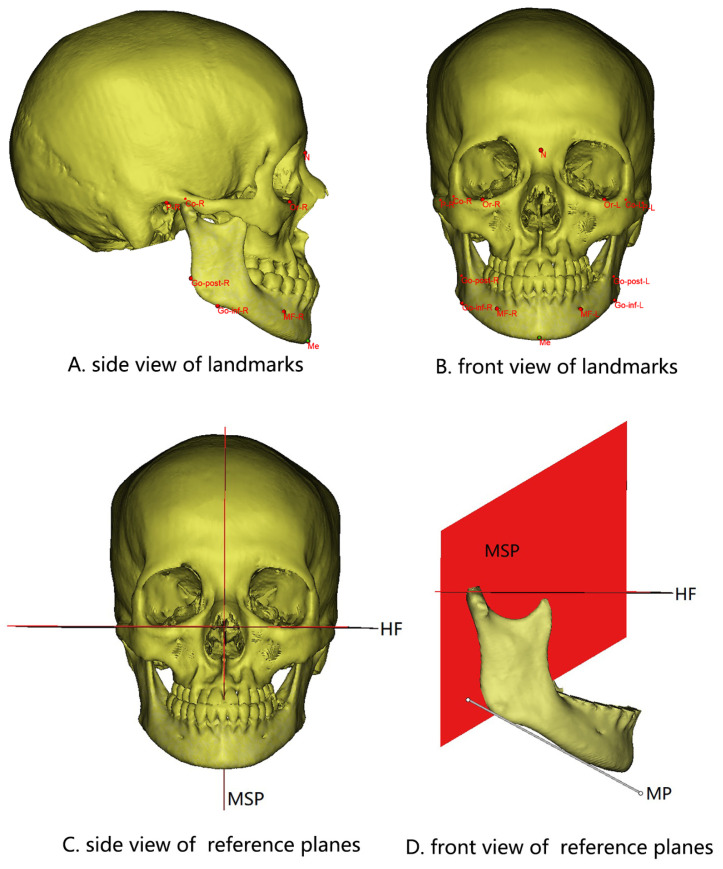
Landmarks (**A**,**B**) and reference planes (**C**,**D**) selected in this study. HF, Frankfort horizontal plane. MP, Tangent to the lowest part of the mandibular inferior border. MSP, Plane passing through N and Ba perpendicular to HF.

**Figure 2:**
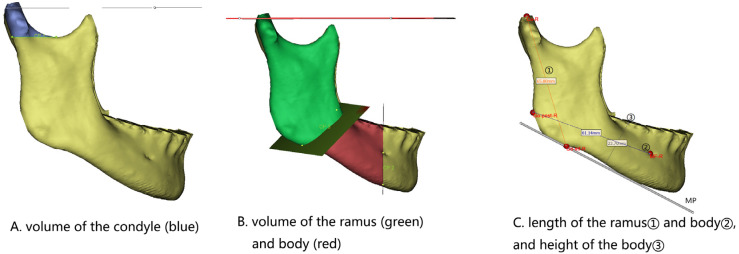
Morphological items evaluated in this study.

### 2.5. Statistical Analysis

Statistical analysis was performed using SPSS software version 22.0 (SPSS, Chicago, IL, USA). Paired *t*-test was applied to compare the symmetry of the same measurement index of the long side and the short side, and a *p*-value < 0.05 was considered statistically significant.

## 3. Results

Fifty patients (11 males and 39 females) were included in this study. The average age was 22.84 ± 3.42 years. 

### 3.1. Symmetric Analysis of Bilateral Mandible before Operation (T0)

The long side of the mandible showed larger condyle volume, larger medial–lateral diameter of the condyle, greater height and volume of the ramus, and greater length and volume of the body. Compared to the short side, the volume and medial–lateral diameter of the condyle, the height and volume of the ramus, and the length and volume of the body were 16.58% (*p* < 0.01), 3.56% (*p* = 0.03), 5.29% (*p* < 0.01), 4.91% (*p* = 0.002), 3.27% (*p* < 0.01) and 2.90% (*p* = 0.026) greater, respectively. There was no difference in the anterior–posterior diameter of the condyle and the body height between the two sides (*p* > 0.05) (Table 2).

### 3.2. Changes in the Long and Short Sides Immediately after Surgery (T1)

For the long side, the ramus volume increased by 275.85 mm^3^ (*p* = 0.001), whereas the body length and volume significantly reduced by 3.64 mm (*p* < 0.01) and 280.83 mm^3^ (*p* = 0.007), respectively. For the short side, the height and volume of the ramus and the body volume tended to increase, while the body length tended to decrease. However, no significant difference was found (Table 3). 

### 3.3. Symmetric Analysis of Bilateral Mandible after Surgery (T1)

After BSSRO, there were still greater ramus height (69.65 ± 5.57 vs. 66.56 ± 5.61 mm, *p* < 0.01) and volume (10,675.08 ± 1915.72 vs. 10,005.36 ± 1957.10 mm^3^, *p* < 0.01) in the long side. Although the difference in the length of the bilateral mandibular body was still statistically significant (*p* = 0.003), the average difference changed from 1.73 mm before surgery to −1.32 mm after surgery, suggesting an overcorrection of the body length by BSSRO. The difference of the bilateral body volume lost statistical meaning (T1, *p* = 0.026; T2, *p* = 0.320). The difference of the bilateral body height did not change after surgery (Table 4). 

### 3.4. Morphological Changes of Bilateral Mandible over Six Months after Surgery (T2)

The morphological data from T2 were compared to those of T1. For the long side of the mandible, the ramus volume and the body volume increased significantly by 591.37 mm^3^ and 501.31 mm^3^ (*p* < 0.01), respectively, and the ramus height and the body height decreased by 0.57 mm (*p* = 0.031) and 0.69 mm (*p* < 0.01), respectively. The morphology of the condyle showed no difference (*p* > 0.05) (Table 5). For the short side of the mandible, the ramus volume and the body volume increased significantly by 347.63 mm^3^ and 634.06 mm^3^ (*p* < 0.01), respectively, and the ramus height and the body height decreased by 0.69 mm (*p* = 0.004) and 0.80 mm (*p* < 0.01), respectively. The morphology of the condyle showed no difference (*p* > 0.05) (Table 5).

The difference between the two sides of the mandible at T1 was also compared to that at T2. The volume of ramus increased from 669.72 ± 1052.14 mm^3^ to 913.46 ± 1152.58 mm^3^ (*p* < 0.05). No significant difference was found for the other items (*p* > 0.05), suggesting a stability of the mandibular morphology after BSSRO (Table 6). 

## 4. Discussion

Relapse after orthognathic surgery can be divided into early relapse and late relapse. Early relapse generally refers to the unexpected displacement of bone fragments within 6–8 weeks after operation. An important cause of late relapse is condylar resorption, which usually occurs 6–17 months after surgery [14]. One of the index of success for orthognathic surgery is the stability of maxillofacial bone. Most of the reported studies have focused on skeletal Class II malocclusion. There are few research studies on mandibular protrusion, especially in patients with asymmetric mandibular prognathism, and most of the available studies have focused on the changes of the condylar angle.

From the results, we found that the measurements of bilateral mandibles had changed, but many of them were not statistically significant. At T3, even if the *p*-values of the changes in ramus height and body height were less than 0.05, the mean values were less than 1 mm, which was not enough to affect the patient’s facial form and occlusion. Therefore, it was not clinically significant.

According to Table 6, at six months or even longer after operation, the symmetry of the mandible had not changed greatly, and only the difference value of the ramus volume increased. We supposed that there was a larger space between the ramus bone blocks on the long side at T1, and the new bone mass was relatively greater. However, the body volume at T2 had no obvious changes. Therefore, it could be proposed that BSSRO has good stability for asymmetric mandibular prognathism. Most studies have reached the same conclusions [15,16].

A portion of patients may have some residual asymmetry of mandible after BSSRO. Our results suggested that the bone mass of the ramus was unbalanced, and it might be the main reason for the residual asymmetry post-operation. Han Lin [17] found that both vertical and transverse discrepancies contributed to the asymmetry of the ramus, so profile plastic surgeries, such as outer bone cortex grinding, could be combined for more ideal outcome.

We also measured the condylar angles in three time periods to further explore the asymmetry of the condylar angles before operation and the changing trends after operation. The results were similar to those of the published literature [18,19,20].

The change in temporomandibular joint (TMJ) after orthognathic surgery has always been a hot topic in research. Ueki et al [21] measured and analyzed the magnitude and direction of occlusal force, as well as the displacement of condyles before and 3–6 months after BSSRO. Their results revealed that the measurement after operation was smaller than that before operation, indicating that the unequilibrium stress of the TMJ had improved. The same conclusion was reached in a study by Shu et al [22]. During this experiment, we noticed that 11 patients appeared to have condylar resorption by observing the spiral CT of the 50 patients at more than 6 months after operation. Moreover, 80 percent occurred in the short side. However, only two patients had a recurrence of more than 1 mm, and it was not obvious. Some scholars have reported that there is a weak to severe correlation between the absorption and the displacement of condyle, that is, the more displacement occurs in the direction of a condyle, the more bone absorption happens on the surface [18]. Absorption of condyle, one of the risk factors for relapse, should be avoided as much as possible.

The bone fragment space was gradually filled with new bone during the six months after BSSRO, so the volumes of the ramus and the body on both sides obviously enlarged. This study showed that the gaps between the bone blocks on both sides of patients with asymmetric mandibular prognathism healed well at one year after BSSRO [23]. During this period, the new bone remodeled in order to adapt to the mandibular morphology.

During operation, for better occlusion, it is necessary to rotate the distal segment of the mandible for some patients. In this study, the internal osseous lamella was below the lower edge of the mandibular body, which indirectly increased the height of the ramus after BSSRO. After a period of remodeling, the inner table was absorbed to adapt to the mandible shape, and the height of the ramus further reduced. We speculated that this might be one of the reasons why the bilateral ramus height decreased at six months after surgery.

In addition, the body height on both sides decreased significantly at T2. Some scholars have also conducted similar studies and found that, whether advance or setback, slight bone resorption might occur above the titanium plate one year after SSRO, which reduces the body height [24].

Some researchers have suggested that the amount of mandibular setback is positively correlated with postoperative relapse [14,25], that is, the greater the mandible setback, the higher the risk of recurrence. However, other researchers have argued against this statement through their research [26]. This study has two limitations. First, the operations were performed by different medical teams and, thus, the surgical experiences might be different. Second, for a more accurate assessment of long-term stability after surgery, the follow-up time could have been longer. In other words, the factors affecting the stability of the mandible in patients with asymmetric mandibular prognathism after BSSRO are not completely clear at present, and more relevant studies are still needed.

## 5. Conclusions

The results of this study suggested good effect and stability of BSSRO on asymmetric mandibular prognathism, except for the correction of the ramus height and volume.

## Figures and Tables

**Table 1:** Definition of the selected landmarks and reference planes.

Landmarks and Reference Planes	Definition
Or, R/L	The most inferior point of the bony orbitale
P, R/L	The most superior point of the external auditory meatus
Ba	Midpoint of the anterior margin of the foramen magnum on the occipital point
N	The junction of the nasal and frontal bones in the midline
Me	The most inferior midpoint of the mandibular symphysis
Go-inf, R/L	The most inferior point on the mandibular angle
Go-post, R/L	The most posterior point on the mandibular angle
Co, R/L	The most superior point of the condylar head
MF, R/L	Mental foramen
J-lat, R/L	The most lateral and deepest point of the curvature at the junction of the mandibular ramus and body
J-med, R/L	The most medial and deepest point of the curvature formed at the junction of the mandibular ramus and body
HF	Frankfort horizontal plane
MSP	Plane passing through N and Ba perpendicular to HF
MP	Tangent to the lowest part of the mandibular inferior border

**Table 2:** Comparison of the mandibular morphology on both sides before surgery (T0) in patients with asymmetric mandibular prognathism.

Measurement	Long Side	Short Side	Difference	*p* (Long Side-Short Side)
Condyle volume (mm^3^)	1991.30 ± 506.22	1708.05 ± 499.37	283.25 ± 380.96	0.000 **
Medial–lateral diameter of condyle (mm)	19.49 ± 2.46	18.82 ± 2.22	0.67 ± 2.12	0.030 *
Anterior–posterior diameter of condyle (mm)	8.25 ± 1.41	8.11 ± 1.66	0.14 ± 1.17	0.398
Ramus height (mm)	69.91 ± 5.81	66.39 ± 5.50	3.51 ± 4.85	0.000 **
Ramus volume (mm^3^)	10,399.23 ± 1919.99	9912.39 ± 1947.23	486.85 ± 1056.21	0.002 **
Body volume (mm^3^)	10,378.98 ± 1775.21	10,086.02 ± 1759.39	292.96 ± 899.77	0.026 *
Body length (mm)	67.24 ± 4.49	65.11 ± 4.82	2.13 ± 2.59	0.000 **
Body height (mm)	26.42 ± 2.73	26.89 ± 2.59	−0.47 ± 1.64	0.051

Paired *t*-test. Data are expressed as Mean ± SD (*n* = 50). * *p* < 0.05, ** *p* < 0.01.

**Table 3:** Comparison of the mandibular morphology on the same side before (T0) and after surgery (T1) in patients with asymmetric mandibular prognathism.

	Measurement	T0	T1	Difference	*p* (T1-T0)
Long Side	Ramus height (mm)	69.91 ± 5.81	69.65 ± 5.57	−0.26 ± 1.59	0.260
	Ramus volume (mm^3^)	10,399.23 ± 1919.99	10,675.08 ± 1915.72	275.85 ± 565.35	0.001 **
	Body volume (mm^3^)	10,378.98 ± 1775.21	10,098.15 ± 1804.49	−280.83 ± 710.88	0.007 **
	Body length (mm)	67.24 ± 4.49	63.60 ± 4.54	−3.64 ± 2.34	0.000 **
	Body height (mm)	26.42 ± 2.73	26.54 ± 2.62	0.12 ± 0.67	0.226
Short Side	Ramus height (mm)	66.39 ± 5.50	66.56 ± 5.61	0.17 ± 1.61	0.458
	Ramus volume (mm^3^)	9912.39 ± 1947.23	10,005.36 ± 1957.10	92.97 ± 564.33	0.250
	Body volume (mm^3^)	10,086.02 ± 1759.39	10,257.40 ± 1815.77	171.38 ± 595.18	0.051
	Body length (mm)	65.11 ± 4.82	64.94 ± 4.62	−0.17 ± 2.11	0.567
	Body height (mm)	26.89 ± 2.59	26.87 ± 2.59	−0.02 ± 0.69	0.833

Paired *t*-test. Data are expressed as Mean ± SD (*n* = 50). ** *p* < 0.01.

**Table 4:** Comparison of the mandibular morphology on both sides after surgery (T1) in patients with asymmetric mandibular prognathism.

Measurement	Long Side	Short Side	Difference	*p* (Long Side-Short Side)
Ramus height (mm)	69.65 ± 5.57	66.56 ± 5.61	3.09 ± 4.68	0.000 **
Ramus volume (mm^3^)	10,675.08 ± 1915.72	10,005.36 ± 1957.10	669.72 ± 1052.14	0.000 **
Body volume (mm^3^)	10,098.15 ± 1804.49	10,257.40 ± 1815.77	−159.25 ± 1121.66	0.320
Body length (mm)	63.60 ± 4.54	64.94 ± 4.62	−1.34 ± 3.06	0.003 **
Body height (mm)	26.54 ± 2.62	26.87 ± 2.59	−0.33 ± 1.75	0.188

Paired *t*-test. Data was expressed as Mean ± SD (*n* = 50). ** *p* < 0.01.

**Table 5:** Comparison of the mandibular morphology on the same side immediately after (T1) and at over 6 months after surgery (T2) in patients with asymmetric mandibular prognathism.

	Measurement	T1	T2	Difference	*p* (T2-T1)
Long Side	Condyle volume (mm^3^)	2040.25 ± 506.47	1989.49 ± 523.03	−50.77 ± 202.40	0.082
	Medial–lateral diameter of condyle (mm)	19.47 ± 2.44	19.73 ± 2.78	0.25 ± 1.09	0.106
	Anterior–posterior diameter of condyle (mm)	8.35 ± 1.42	8.38 ± 1.43	0.03 ± 0.63	0.739
	Ramus height (mm)	69.65 ± 5.57	69.08 ± 6.05	−0.57 ± 1.82	0.031 *
	Ramus volume (mm^3^)	10,675.08 ± 1915.72	11,266.45 ± 1987.58	591.37 ± 688.78	0.000 **
	Body volume (mm^3^)	10,098.15 ± 1804.49	10,599.45 ± 1893.86	501.31 ± 762.77	0.000 **
	Body length (mm)	63.60 ± 4.54	63.99 ± 4.27	0.39 ± 1.37	0.052
	Body height (mm)	26.54 ± 2.62	25.85 ± 2.90	−0.69 ± 1.08	0.000 **
Short Side	Condyle volume (mm^3^)	1744.72 ± 515.87	1681.99 ± 569.73	−62.73 ± 235.78	0.066
	Medial–lateral diameter of condyle (mm)	18.76 ± 2.17	18.81 ± 2.39	0.04 ± 0.82	0.724
	Anterior–posterior diameter of condyle (mm)	8.18 ± 1.74	8.05 ± 1.78	−0.13 ± 0.77	0.230
	Ramus height (mm)	66.56 ± 5.61	65.87 ± 5.67	−0.69 ± 1.62	0.004 **
	Ramus volume (mm^3^)	10,005.36 ± 1957.10	10,352.99 ± 1895.42	347.63 ± 699.86	0.001 **
	Body volume (mm^3^)	10,257.40 ± 1815.77	10,891.46 ± 2164.02	634.06 ± 822.44	0.000 **
	Body length (mm)	64.94 ± 4.62	64.92 ± 4.81	−0.02 ± 1.51	0.937
	Body height (mm)	26.87 ± 2.59	26.07 ± 3.00	−0.80 ± 1.20	0.000 **

Paired *t*-test. Data are expressed as Mean ± SD (*n* = 50). * *p* < 0.05, ** *p* < 0.01.

**Table 6:** Comparison of the difference between the two sides of the mandible within 2 weeks after surgery (T1) and at over 6 months after surgery (T2) in patients with asymmetric mandibular prognathism.

Measurement	T1	T2	*p*
Ramus height difference (mm)	3.09 ± 4.68	3.21 ± 4.70	0.717
Ramus volume difference (mm)	669.72 ± 1052.14	913.46 ± 1152.58	0.019 *
Body volume difference (mm^3^)	−159.25 ± 1121.66	−292.01 ± 1224.09	0.283
Body length difference (mm)	−1.34 ± 3.06	−0.93 ± 3.23	0.130
Body height difference (mm)	−0.33 ± 1.75	−0.22 ± 1.76	0.469
Condyle volume difference (mm^3^)	295.53 ± 415.80	307.49 ± 497.44	0.723
Medial–lateral diameter of condyle difference (mm)	0.71± 2.05	0.92 ± 2.39	0.165
Anterior–posterior diameter of condyle difference (mm)	0.16 ± 1.34	0.33 ± 1.38	0.273

Paired *t*-test. Data are expressed as Mean ± SD (*n* = 50). * *p* < 0.05.

## Data Availability

The data presented in this study are available on request from the corresponding author. The data are not publicly available due to privacy.
